# Response of *Macrobrachium rosenbergii* to Vegetable Oils Replacing Dietary Fish Oil: Insights From Antioxidant Defense

**DOI:** 10.3389/fphys.2020.00218

**Published:** 2020-03-13

**Authors:** Cunxin Sun, Bo Liu, Qunlan Zhou, Zhe Xiong, Fan Shan, Huimin Zhang

**Affiliations:** ^1^Key Laboratory of Freshwater Fisheries and Germplasm Resources Utilization, Ministry of Agriculture, Freshwater Fisheries Research Center, Chinese Academy of Fishery Sciences, Wuxi, China; ^2^Wuxi Fisheries College, Nanjing Agricultural University, Wuxi, China

**Keywords:** vegetable oil, fish oil, antioxidant defense, NF-κB/NO signal pathway, *Macrobrachium rosenbergii*

## Abstract

The study was conducted to evaluate the effects of fish oil replacement by vegetable oils on growth performance, histology, and antioxidant capacity of *Macrobrachium rosenbergii*. Three isonitrogenous and isoenergetic diets were formulated with different lipid sources included. DFO diet contained 6% fish oil, whereas DSO and DRO diets included 6% soybean oil and rapeseed oil (RO) as alternatives for fish oil, respectively. Prawns were fed thrice daily for 8 weeks. The results showed that prawns in DFO group showed significantly lower final weight, weight gain ratio, and specific growth rate (SGR), but higher feed intake and feed coefficient ratio than those in DSO and DRO groups. In hepatocellular ultrastructure, malformed and atrophic nucleus and higher apoptosis ratio were observed in DFO group. In addition, levels of haemolymph proinflammatory cytokines, activities of anti-superoxide anion, inducible-type NO-synthase (iNOS) and content of nitric oxide, and hepatopancreas NF-κB signal pathway gene expression in DFO group increased markedly compared to those of DSO and DRO groups. The results suggested that vegetable oils, such as soybean oil and RO might be the better lipid sources in diets for *Macrobrachium rosenbergii* than fish oil, it may be attributed to modified oxidative status induced by NF-κB-NO signal pathway.

## Introduction

Marine fish oil is commonly used as the main lipid ingredient in aquafeeds, especially for rapid-growing fish and crustacean species which require LC-PUFA as EFA, such as (20:5n-3, EPA), docosahexaenoic acid (22:6n-3, DHA) and arachidonic acid (20:4n-6, AA). Providing an EFA balanced diet is not only essential for maintaining growth and food utilization, but also for immunity and resistance to oxidative stress of aquatic animals (Tocher et al., 2010).

Nevertheless, the reduction in global fish oil supply has forced this industry to use alternative fat sources in aquafeeds. The relative richness of plants makes vegetable oils a good alternative and sustainable source. Vegetable oils contain abundant C18 PUFA, such as (18:1n-9, OA), (18:2n-6, LA) and (18:3n-3, LNA), but are low in n-3 LC- PUFA (Turchini et al., 2010). Many freshwater teleosts can potentially convert LA to AA by desaturating and elongating via enzymatic pathways (Bell and Tocher, 2009), in which Δ6 and Δ5 fatty acyl desaturases (Fad) play a pivotal role. However, this capability in crustaceans is currently unknown. Previously, expression of Δ6 Fad has been conducted on euryhaline and freshwater crustaceans, Chinese mitten crab (*Eriocheir sinensis*) and Pacific white shrimp (*Litopenaeus vannamei*) (Yang et al., 2013; Chen K. et al., 2017). Δ12 and Δ15 Fads have been detected in some species of plants and invertebrates for n-3 and n-6 PUFA synthesis through LA (National Research Council [NRC], 2011). In addition, *M. rosenbergii* is unable to synthesize 18:2n-6 or 18:3n-3, but elongates and desaturates 18:2n-6 to 20:2n-6, 20:3n-6 and 18:3n-3 to 22:6n-3 (Reigh and Stickney, 1989). *M. rosenbergii* is also unable to synthesize either 20:4n-6 or 20:5n-3 from shorter chain fatty acids, but the requirements of the two fatty acids are small amount (Del Rosario González-Baró and Pollero, 1998; National Research Council [NRC], 2011). *M. rosenbergii* can use OA efficiently (Querijero et al., 1997). Thus, *M. rosenbergii* might have the capacity to use vegetable oils rich in C18 fatty acids.

Accordingly, single or blend of vegetable oils have been tested in *M. rosenbergii*, such as soybean oil, sunflower oil, coconut oil, palm oil, castor oil, corn oil, and RO (canola oil), and some of them (soybean oil, sunflower oil, and RO) have a comparable or better effects on growth performance compared with fish oil (Kamarudin and Roustaian, 2002; Kim et al., 2013; Kangpanich et al., 2017). Among them, soybean oil and RO have great potential to be used in the feed of *M. rosenbergii* because of the considerable contents of OA and LA, respectively, (National Research Council [NRC], 2011), and the abundant availability makes these two vegetable oils more preferable for low cost aquafeed production. However, some reports pointed out that vegetable oils could reduce the survival rate and post-larval production and of shrimps because of the fatty acid profiles (Petriella et al., 1984; Kamarudin and Roustaian, 2002).

Numerous studies indicate that the imbalance of dietary fatty acids can induce inflammation and oxidative stress, a reaction of immune system response to external physical, chemical stimuli or bacterial infiltration. However, chronic or excessive stress reactions could lead to inflammatory diseases. During an intracellular signal cascade, Rel/NF-κB family makes an important impact in the activation of the immunological factors involved with inflammation and apoptosis (Dixit and Mak, 2002; Hoffmann and Reichhart, 2002; Hayden and Ghosh, 2008).

There are also two NF-κB family signal pathways (Relish or Dorsal pathway) in *Drosophila melanogaster*, a representative invertebrate, and the two signal pathways also contribute to anti-bacterial and immune responses (De Gregorio et al., 2002; Hoffmann and Reichhart, 2002). Dorsal/Dif interacts with Cactus, an IκB-related inhibitor, but in response to the degradation of Cactus, Dorsal/Dif was activated by Toll receptor signaling and translocated to the nucleus to interact with target gene regulatory sites (Busse et al., 2007). In *Drosophila*, the Toll-Dorsal pathway plays a core role in response to infection by microorganisms and viruses through the synthesis of antibacterial peptides (AMPs) (Valanne et al., 2011). Similar with vertebrate p105, Relish is triggered by the IMD pathway in response to infection by activating the gene expression of AMPs in *Drosophila* (Lemaitre and Hoffmann, 2007). Also, some key genes in Toll and IMD pathway of *M. rosenbergii* have been reported, but most research focuses on viral preventing and controlling (Srisuk et al., 2014; Shi et al., 2015; Huang et al., 2016). In mammals, research has revealed that NF-κB can regulate oxidative stress by increasing iNOS activity and subsequently inducing nitric oxide (NO) synthesis (Janssen-Heininger et al., 2016; Marinello et al., 2019). NO can be combined with oxygen radicals to reduce oxidative stress (Aktan, 2004). However, less research has focused on oxidative stress regulated by dietary lipid via NF-κB/NO signal pathway in shrimp. Therefore, the signaling pathways involved in NF-κB and oxidative stress regulation in shrimp is an area for further research.

The giant freshwater prawn, *M. rosenbergii*, has been cultured in tropical and subtropical regions for a long time. This variety has excellent cultivation characteristics such as fast growth, high market demand, strong resistance to stress, and adaptation to euryhaline environments (Moffitt and Cajas-Cano, 2014). It supports the livelihood in low-income communities in many parts of world and strengthens the use of marginal inland waters to produce high-value goods. However, high feed cost and serious disease problem in intensive aquaculture limits the industrial development (Miao and Ge, 2002; Fu et al., 2012). Thus, the objective of the present study was to evaluate the efficacy of vegetable oils replacing dietary fish oil on growth and antioxidative capacity of *M. rosenbergii.*

## Materials and Methods

### Animal Ethics

The care and use of animals followed Animal Research Institute Committee guidelines of Nanjing Agricultural University, China. This study has been approved by the Committee of the Animal Research Institute of Nanjing Agricultural University, China [permit number: SYXK (Su) 2011-0036].

### Experimental Ingredients and Diets

Three isonitrogenous (40.9% crude protein) and isoenergetic (16.3 MJ kg^–1^ gross energy) diets were formulated in this study. The control diet included 6% fish oil (DFO), while fish oil was substituted with soybean oil and rapeseed oil in the test diets (DSO and DRO). Formulation and proximate composition of the experimental diets is presented in Table 1. Fish meal, casein and gelatin served as protein sources; fish oil, soybean oil, and RO were supplemented as lipid sources; α-starch and dextrin were used as carbohydrate source. Fatty acid composition of the feeds is shown in Table 2.

**TABLE 1:** Formulation and proximate composition of the experimental diets.

	DFO	DSO	DRO
**Ingredients%**			
Fish meal^1^	22.00	22.00	22.00
Casein^2^	24.00	24.00	24.00
Gelatin^1^	6.00	6.00	6.00
α-starch^3^	20.00	20.00	20.00
Dextrin^3^	5.00	5.00	5.00
Fish oil^1^	6.00	0.00	0.00
Soybean oil^1^	0.00	6.00	0.00
Rapeseed oil^1^	0.00	0.00	6.00
Microcystalline cellulose^4^	5.00	5.00	5.00
Carboxymethyl cellulose^4^	3.00	3.00	3.00
Attapulgite^1^	1.53	1.53	1.53
Soybean lecithin (50%)^1^	2.00	2.00	2.00
Cholesterol^1^	0.30	0.30	0.30
Eclosion hormone^5^	0.10	0.10	0.10
DMPT^5^	0.05	0.05	0.05
choline chloride^5^	1.00	1.00	1.00
Vitamin premix^5^	1.00	1.00	1.00
Vitamin E^1^	0.02	0.02	0.02
Mineral premix^5^	1.00	1.00	1.00
Calcium dihydrogen phosphate^1^	2.00	2.00	2.00
**Proximate composition%**			
Crude protein	40.90	40.60	41.10
Crude lipid	8.86	8.55	8.68
Gross energy (MJ kg^–1^)	16.30	16.30	16.30

*^1^Obtained from Wuxi Tongwei feedstuffs Co., Ltd., Wuxi, China. ^2^Obtained from Hulunbeier Sanyuan Milk Co., Ltd., Inner Mongolia, China. ^3^Obtained from Yinhe Dextrin Co., Ltd., Zhengzhou, China. ^4^Obtained from Yifeng Food Additives Co., Ltd., Shanghai, China. ^5^Obtained from Jiangsu Fuyuda Food Products Co., Ltd., Yangzhou, China.*

**TABLE 2:** Fatty acid composition of the feed (%).

	DFO	DSO	DRO
C12:0	0.09	0.07	0.15
C14:0	2.34	1.06	1.70
C15:0	0.41	0.13	0.16
C16:0	29.78	14.54	10.58
C16:1n-9	10.81	1.96	1.72
C17:0	1.02	0.23	0.16
C18:0	5.54	4.43	3.32
C18:1n-9	23.95	22.94	43.86
C18:2n-6	4.55	44.69	18.82
C18:3n-6	0.49	0.07	0.06
C18:3n-3	1.65	5.17	5.02
C20:0	0.24	0.35	0.56
C20:1n-9	0.60	0.31	3.14
C20:2n-9	0.07	0.05	0.20
C20:3n-3	0.19	0.04	0.05
C20:4n-6	0.92	0.18	0.25
C20:5n-3	12.43	1.61	2.12
C22:0	0.00	0.00	0.31
C22:1n-13	0.29	0.15	5.20
C22:3n-3	0.03	0.02	0.06
C22:4n-3	0.12	0.05	0.06
C22:5n-3	0.51	0.34	0.34
C22:6n-3	4.00	1.64	2.15
n-6 PUFA	5.11	44.81	19.08
n-3 PUFA	19.84	9.03	10.06

All the ingredients were ground through a 60-mm mesh. The fine powder was carefully weighed, then 6% oil (w/w) and 30% water (w/w) were added to the mixture that was further blended to ensure homogeneity. A Laboratory pelletizer (Guangzhou Huagong Optical Mechanical & Electrical Technology CO. LTD, Guangzhou, China) was used for the pelletizing process. Feed pellets (1 mm and 1.5 mm diameter) were produced for prawns in different growth periods. After drying in feed dryer, the feeds were offered to prawns.

### Shrimps and the Feeding Trial

Juvenile prawns were provided by Zhejiang Southern Taihu Lake Freshwater Aquatic Seed Industry Co., Ltd., (Huzhou, China). After 2 weeks of acclimation, prawns of similar size (0.24 ± 0.001 g) were randomly distributed into 9 concrete tanks (2.0 × 2.0 × 0.8 m) at a density of 50 prawns per tank. Three experimental diets were randomly allotted to prawns in triplicate tanks.

All prawns were fed three times daily at 7:00, 12:00, and 17:30 for 56 days, and feeding amount was 2–5% of body weight. Feces and molts were removed by siphoning. During the feeding trial, the average water temperature was 30 ± 0.4°C; continuous aeration was supplied to each tank to maintain the dissolved oxygen above 50 mg/L; pH was 7.6–8.0, and total ammonia nitrogen level was above 0.02 mg/L, Total ammonia nitrogen and nitrite were kept <0.2 and 0.005 mg/L, respectively.

### Sample Collection

At the end of the feeding period, prawns were fasted for 24 h to empty the digestive tract. Before sampling, the prawns were placed in the mixture of water and ice. The hemolymph from 5 prawns was randomly sampled per tank using heparinized syringes. Hemolymph samples were collected into anticoagulation tubes, then centrifuged at 2000 × *g* 4°C for 10 min. Hepatopancreas samples were stored in 4% paraformaldehyde for apoptosis measurement and 2.5% glutaraldehyde for ultrastructure study, respectively. The remainder of the hepatopancreas of each prawn was stored at −80°C for subsequent analysis of gene expression.

### Proximate and Fatty Acid Analysis

Diets were analyzed for proximate composition. Crude protein (nitrogen × 6.25) was determined by the Kjeldahl method using an Auto Kjeldahl System (2300; FOSS Tector, Hoganas, Sweden); crude lipid was determined by ether extraction using an Auto Soxtec System (2050; FOSS Tector); gross energy was determined by an adiabatic bomb calorimeter (PARR 1281, United States).

The total lipids of experimental diets were extracted according to the Bligh–Dyer’s method (Bligh and Dyer, 1959), and the obtained total lipids were weighed and stored at −20°C until used. Afterward, fatty acids were converted to their methyl esters for Gas Chromatographic (GC) analysis according to Nasopoulou et al. (2011). Agilent 5890 series II GC (Agilent Technologies, Santa Clara, CA, United States) was used for GC analysis of fatty acids.

### Ultrastructure Study

Electron microscopy samples were fixed with 2.5% glutaraldehyde for 24 h, then fixed with 1% osmium tetroxide for 1 h and stored at 4°C. The sections were embedded in epoxy resin Epon 812, cut into70-nm-thick sections by RMC PowerTomeXL microtome, stained with uranyl acetate and lead citrate, and examined under a transmission electron microscope (Hitachi H-7650, Tokyo, Japan).

### Apoptosis Detection

Hepatocyte apoptosis was determined according to Lu et al. (2013); the terminal deoxynucleotidyl TUNEL assay followed the protocol of Apoptosis Detection Kit (Nanjing Jian-Cheng Bioengineering Institute, China). The tissue sections were deparaffinized, rehydrated, and treated with protein digestion buffer at room temperature for 10–15 min, followed by incubation of biotinylated nucleotide mix with the working-strength TDT at 37°C for 60 min. The reactions were terminated by immersing the slides in 2 × saline sodium citrate at room temperature for 10–15 min. Each slide was incubated with 100 mL of dimethylaminoazobenzene (DAB) and developed until a light brown background appears. The slides were mounted in an aqueous mounting medium followed by the examination under a light microscope. The positive cell nucleus was dyed brown–yellow. The DNase1-treated tissue was used as the positive control. The reaction without TDT enzyme was used as the negative control.

### Levels of Proinflammatory Cytokines

Tumor necrosis factor-α (TNF-α), interferon-γ (INF-γ), interleukin-1 (IL-1), and interleukin-6 (IL-6) in hemolymph were measured using enzyme-linked immunosorbent assay (ELISA) kits (Shanghai Enzyme-linked Biotechnology Co., Ltd., Shanghai, China) following the manufacturer’s protocols.

### iNOS, NO and Anti-Superoxide Anion in Hemolymph

iNOS activity, NO content, and ASA activity in hemolymph was detected using a commercially available assay kit (Nanjing Jiancheng Bioengineering Institute, China) according to the manufacturer’s instructions.

### RNA Isolation and RT-qPCR Analysis

Total RNA was isolated using RNAiso Plus (Takara Co., Ltd., Japan), and then purified with RNase-Free DNase (Takara Co., Ltd., Japan) to avoid genomic DNA amplification. Purity and concentration of RNA were measured using a NanoDrop (DN-1000, Thermo Fisher Scientific, United States). After normalizing the concentration of the RNA samples, cDNA was generated from 500 ng DNase-treated RNA using ExScriptTM RT-PCR kit according to the manufacturer’s directions (Takara Co., Ltd., Japan).

The cDNA samples were analyzed using a real-time quantitative detector (BIO-RAD, United States) with the SYBR Green II Fluorescence Kit (Takara Co., Ltd., Japan). The fluorescent qPCR reaction solution consisted of 10 μL SYBR^®^ premix Ex Taq^TM^, 0.4 μL ROX Reference Dye II, 0.4 μL PCR forward primer (10 μM), 0.4 μL PCR reverse primer (10 μM), 2.0 μL RT reaction (cDNA solution), and 6.8 μL dH2O. All RT-qPCR primers were designed using the Primer 5 software and listed in Table 3. The thermal profile was 95°C for 30 s, followed by 40 cycles of 95°C for 5 s and 60°C for 30 s, followed by a melt curve analysis of 15 s from 95 to 60°C, 1 min for 60°C, and then up to 95°C for 15 s. Control reactions were conducted with non-reverse transcribed RNA to determine the level of background or genomic DNA contamination. In all cases, genomic DNA contamination was negligible. β-actin was selected as the housekeeping gene to normalize our samples because of its stable expression in the present study. qPCR reactions were carried out in triplicates of each sample. Values for the threshold (CT) from the treated and control tissue templates were compared, and the 2^–△△CT^ method was used as the relative quantification calculation method (Livak and Schmittgen, 2001).

**TABLE 3:** Sequences of the primers used in the study.

Gene	GenBank acc. no.	Primer sequences (5′-3′)	Length (bp)	Amplicon length (bp)	References
Relish	KR827675.1	GATGAGCCTTCAGTGCCAGA	20	238	
		CCAGGTGACGCCATGTATCA	20		
Dorsal	KX219631.1	TCAGTAGCGACACCATGCAG	20	200	
		CGAGCCTTCGAGGAACACTT	20		
IκBα	HQ668091.1	AATCATACCGGAAGGACGGCGTTA	24	133	Arockiaraj et al., 2012
		TCACGGGTCTGGTTAATTGGGTCA	24		
IMD		CGACCACATTCTCCTCCTCCC	21	184	Shi, 2016
		TTCAGTGCATCCACGTCCCTC	21		
Toll	KX610955.1	TTCGTGACTTGTCGGCTCTC	20	227	
		GCAGTTGTTGAAGGCATCGG	20		
β-actin	AY651918.2	TCCGTAAGGACCTGTATGCC	20	96	Liu et al., 2012
		TCGGGAGGTGCGATGATTTT	20		

### Calculations and Statistical Analysis

The growth parameters were calculated as follows, where W_0_ and W_*t*_ are initial and final body weight.

Weight gain rate (WGR,%)=(W-tW)0×100/W.0

Specific growth rate (SGR,%day)-1=(LnW-tLnW)0×100/day.

FCR = feed⁢consumption⁢(g)/Weight⁢gain⁢(g).

Relative feed intake (RFI,%/day)= 100×totalamountofthefeedconsumed×2/[(W+0W)t×days]

Data were subjected to one-way analysis of variance (ANOVA) to investigate the growth performance, hemolymph parameters, and mRNA expression, after testing the homogeneity of variances with the Levene test. If significant (*P* < 0.05) differences were found, Tukey’s multiple range test was used to compare the means. Analyses were performed using the SPSS program version 16.0 (SPSS Inc., Michigan Avenue, Chicago, IL, United States) for Windows. All data were presented as means ± S.E.M (standard error of the mean).

## Results

### Growth Performance

As shown in Table 4, at the end of the feeding trial, final weight, WGR and SGR in DSO and DRO were significantly higher than those in DFO (*P* < 0.05), while no significant difference was observed between DSO and DRO (*P* > 0.05). Significant differences in RFI and FCR were only found between DFO and DSO (*P* < 0.05).

**TABLE 4:** Growth performance and feed utilization of *Macrobrachium rosenbergii* fed with different dietary lipid sources.

	IW	FW	WGR	SGR	RFI	FCR
DFO	0.24 ± 0.001	9.4 ± 0.17^a^	3859.7 ± 112.0^a^	5.93 ± 0.05^a^	5.88 ± 0.03^b^	1.92 ± 0.01^b^
DSO	0.24 ± 0.001	12.4 ± 0.21^b^	4963.0 ± 61.6^b^	6.33 ± 0.02^b^	4.55 ± 0.37^a^	1.47 ± 0.12^a^
DRO	0.24 ± 0.003	11.9 ± 0.09^b^	4772.5 ± 84.7^b^	6.27 ± 0.03^b^	5.22 ± 0.23^ab^	1.68 ± 0.07^ab^

*IW, initial weight; FW, final weight; WGR, weight gain rate (%) = (W_*t*_–W_0_) × 100/W_0_; SGR, specific growth rate (% day_–__1_) = (LnW_*t*_–LnW_0_) × 100/day; FCR, feed conversion ratio = feed consumption (g)/weight gain (g); RFI, relative feed intake (%/day) = 100 × total amount of the feed consumed × 2/[(W_0_ + W_*t*_) × days]. Values are means ± SEM (*n* = 3). Means in the same column with different letters indicate significant differences (*P* < 0.05).*

### Ultrastructure of Hepatocytes

**FIGURE 1:**
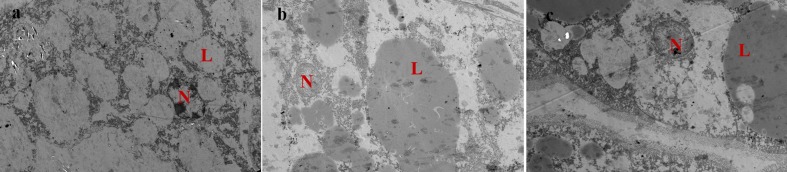
Transmission electron microscope images of *Macrobrachium rosenbergii* hepatocyte ultrastructure (1200 ×). **(a)** Prawns fed diet including 6% fish oil (DFO); **(b)** Prawns fed diet including 6% soybean oil (DSO); **(c)**: Prawns fed diet including 6% rapeseed oil (DRO). N: nucleus; L: lipid droplet.

As shown in Figure 1, hepatopancreas of prawns fed DSO and DRO diets showed normal ultrastructure (Figures 1b,c). In these hepatocytes, the nucleus was round and the nucleolus was visible, and the lipid droplets were big and plump. However, in DFO (Figure 1a), the nucleus was malformed and atrophic; the lipid droplets became smaller and increased in number than those in the other groups.

### Hepatocyte Apoptosis

**FIGURE 2:**
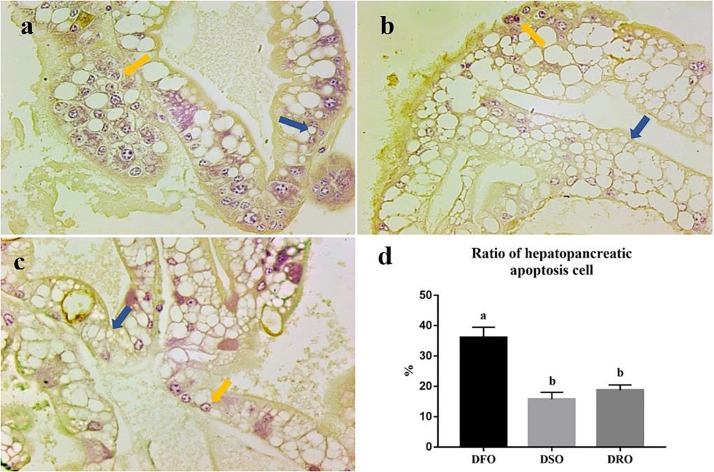
Hepatocyte apoptosis of *Macrobrachium rosenbergii*. **(a)** Prawns fed diet including 6% fish oil (DFO); **(b)** Prawns fed diet including 6% soybean oil (DSO); **(c)** Prawns fed diet including 6% rapeseed oil (DRO); **(d)** Ratio of hepatopancreatic apoptosis cell (*n* = 3). Blue arrow: normal cells; yellow arrow: apoptotic cells.

Apoptosis of hepatopancreatic cells was evaluated by TUNEL assay. The cells with stained brown nuclei were considered in a state of apoptosis (Figure 2) and were counted to calculate hepatopancreatic apoptosis cell ratio. Three paraffin sections were used for TUNEL assay each group. In prawns fed DFO diets, apoptotic cells were about 36% of total hepatocytes (Figure 2a). In the hepatopancreas from prawns fed DSO or DRO diets, the apoptotic cells were about 10–20% of total hepatocytes (Figures 2b,c). Thus, TUNEL positive hepatocytes of prawns fed DFO diet were significantly more than those fed DSO and DRO diets (*P* < 0.05) (Figure 2d).

### Proinflammatory Cytokines in Hemolymph

**FIGURE 3:**
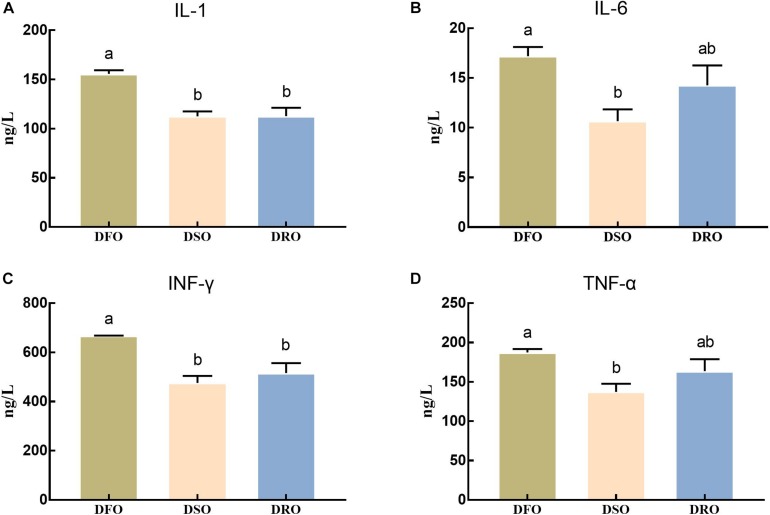
Levels of proinflammatory cytokines in hemolymph. DFO: prawns fed diet including 6% fish oil; DSO: prawns fed diet including 6% soybean oil; DRO: prawns fed diet including 6% rapeseed oil. Values are means ± SEM (*n* = 15). **(A)** IL-1 concerntration in hemolymph; **(B)** IL-6 concerntration in hemolymph; **(C)** INF-γ concerntration in hemolymph; **(D)** TNF-α concerntration in hemolymph. Columns with different letters indicate significant differences (*P* < 0.05).

The levels of inflammatory factors in hemolymph were shown in Figure 3. The levels of IL-1 and INF-γ in DSO and DRO showed significant differences compared with those in DFO (*P* < 0.05), but no significant differences were found between DSO and DRO (*P* > 0.05). The levels of IL-6 and TNF-α in DSO group showed significant difference compared with those in DFO group (*P* < 0.05), but there was no significant difference between the other two pairs (*P* > 0.05).

### Hemolymph Oxidative Status

**FIGURE 4:**
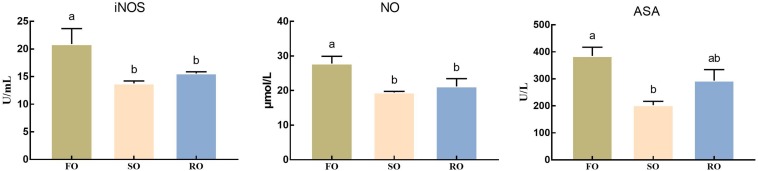
Hemolymph oxidative status of *Macrobrachium rosenbergii.* DFO: prawns fed diet including 6% fish oil; DSO: prawns fed diet including 6% soybean oil; DRO: prawns fed diet including 6% rapeseed oil; ASA: arachidonic acid; iNOS: inducible-type NO-synthas; NO: nitric oxide. Values are means ± SEM (*n* = 15). Columns with different letters indicate significant differences (*P* < 0.05).

As shown in Figure 4, the activity of iNOS and level of NO in hemolymph in DFO fed prawns were significantly higher than those in DSO and DRO fed groups (*P* < 0.05). ASA activity in DFO fed prawns was significantly higher than that in DSO fed group (*P* < 0.05), while no marked difference was observed between DFO and DRO groups (*P* > 0.05).

### Gene Expression in NF-κB Signal Pathway

**FIGURE 5:**
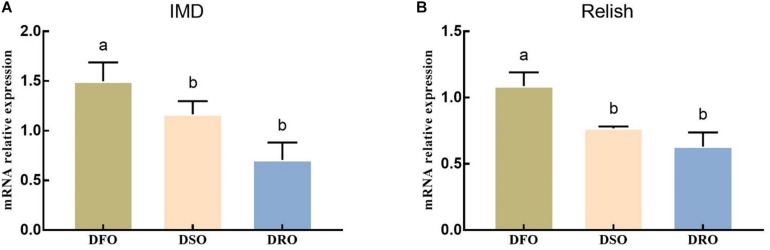
Gene expression of IMD-Relish signal pathway in hepatopancreas. DFO: prawns fed diet including 6% fish oil; DSO: prawns fed diet including 6% soybean oil; DRO: prawns fed diet including 6% rapeseed oil. Values are means ± SEM (*n* = 15). **(A)** The expression of IMD in hepatopancreas; **(B)** the expression of Relish in hepatopancreas. Columns with different letters indicate significant differences (*P* < 0.05).

The mRNA expression of IMD and Relish showed a similar pattern when the prawns were fed different lipid sources (Figure 5). Significant differences were found between DFO and DRO in mRNA expression of IMD (*P* < 0.05), while significant differences were found between DFO and DSO in Relish expression (*P* < 0.05).

**FIGURE 6:**
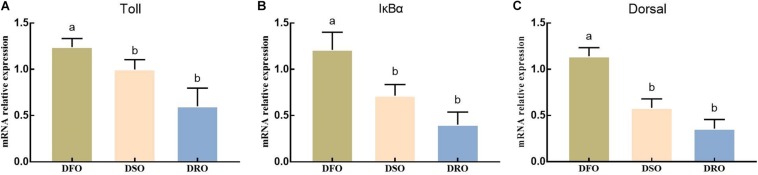
Gene expression of Toll-Dorsal signal pathway in hepatopancreas. DFO: prawns fed diet including 6% fish oil; DSO: prawns fed diet including 6% soybean oil; DRO: prawns fed diet including 6% rapeseed oil. Values are means ± SEM (*n* = 15). **(A)** The expression of Toll in hepatopancreas; **(B)** the expression of IκBα in hepatopancreas; **(C)** the expression of Dorsal in hepatopancreas. Columns with different letters indicate significant differences (*P* < 0.05).

As shown in Figure 6, lipid sources showed significant effects on the mRNA expression of Toll, IκBα and Dorsal (*P* < 0.05). DFO showed the highest expression on Toll, IκBα and Dorsal, significant difference were found between DFO and DRO (*P* < 0.05).

## Discussion

With the increase of vegetable oil production, the prices of multifarious products in some regions have stabilized and even declined (Bell et al., 2004). Hence, in the present study, vegetable oils as candidate to replace fish oil was examined. The results showed that fish oil could be completely replaced by soybean oil or RO in giant freshwater prawn diets for an 8-week feeding period, and even these two vegetable oils showed better growth promoting effects than fish oil. Similar results were also found in some previous studies that showed the viability of replacing dietary fish oil with soybean oil completely (Piedecausa et al., 2007). In this study, the DFO diet had the highest level of PUFA, such as EPA and DHA, while the DSO and DRO diets were rich in LA and OA. The demand for dietary EFA varies widely among different species. In most freshwater fish species, the demands of EFA can be satisfied by C18 PUFA (18:3n-3, 18:2n-6), while C18 PUFA cannot be used to synthetize the EFA in marine fish (Tocher et al., 2010). According to National Research Council [NRC] (2011), the EFA requirements of *M. rosenbergii* are 0.075% (DHA) and 0.08% (AA). According to the dietary fatty acid composition in the present study, all the experimental diets included with 22% fish meal could satisfy the demands of the LC-PUFA. Growth inhibition in DFO group might be attributed to the dietary excess n-3 LC-PUFA (1.10% EPA, 0.35% DHA) rather than optimum demands of *M. rosenbergii.* Similar results on fish oil replacement by vegetable oils in *M. rosenbergii* also showed that vegetable oils had a better effect on growth improvement than fish oil when the basal diet included 9.8–35% fish meal (Kim et al., 2013; Kangpanich et al., 2017). Inverse result was observed when no fish meal was incorporated in basal diet (Muralisankar et al., 2014). Thus, 10% dietary fish meal could supply enough amount of LC-PUFA for *M. rosenbergii.* It was supported by the data that showed excess n-3 HUFA can cause poor growth performance, hepatic pathology, and blood lipid peroxidation in grass carp (*Ctenopharyngodon idella*) (Du et al., 2010), growth restriction and high mortalities in Russian sturgeon (*Acipenser gueldenstaedtii*) (Zhu et al., 2017), and high mortalities and muscular lesions in European sea bass (*Dicentrarchus labrax*) (Betancor et al., 2011). It was further supported by the fact that only vegetable oil or mix of vegetable oil and fish oil was incorporated in the commercial diet included with fish meal of *M. rosenbergii*, but the ratio of fish oil decreased with the increasing proportion of dietary fish meal (Tidwell et al., 1993; Radhakrishnan et al., 2014; Banerjee and Khemundu, 2019).

The histological examination is considered as the gold standard of liver damage detection. Compared with DFO, significant alterations in the ultrastructure of hepatopancreas were found in DSO and DRO, in which lipid droplets were plump and bigger, as well as the nucleus was clear. The atrophy of the organelles in DFO group might be atributted to the excess LC-PUFA in fish oil. Previous studies had evaluated that excess dietary unsaturated fatty acids could cause functional damage to cells through lipid peroxidation (Matés et al., 1999), which could increase levels of reactive oxygen species, attenuate the body antioxidant defenses, and introduce liver injury (Atalay et al., 2000; Sanders et al., 2004; Nieto, 2007). The oxidant status is the decisive factor of most kinds of steatosis hepatis (Tariq et al., 2014). Additionally, many studies indicate that higher dietary soybean oil levels could induce lipid deposition in the liver of fish (Li et al., 2016), which is similar with the result of the present study. This conclusion is further supported by the studies that show dietary LA can increase lipid deposition both in the livers of mammals and fish (Pérez-Matute et al., 2007; Muhlhausler and Ailhaud, 2013). This may be attributed to vegetable oils that increase the key enzyme activies associated with fatty acid synthesis.

Moreover, hepatocyte apoptosis is often associated with hepatopancreas lipid peroxidation, and the evaluation of apoptosis is considered as a novel biomarker of disease severity of liver in human (Wieckowska et al., 2006). In this study, DFO-fed prawns were significantly TUNEL-positive compared to those in the other groups. The steatotic hepatocyte appears to be more susceptible to apoptosis. A previous study reported that fish oil can modulate the redox environment by increasing apoptosis, which is mediated by ROS (Sanders et al., 2004), but other reports concluded fish oil has the ameliorating effects of oxidative stress and apoptosis (Myhrstad et al., 2014; Hong et al., 2015). The contradiction might be due to the dose because excess n-3 HUFA led to lipid peroxidation and oxidative stress in the present study.

Cytokines including the interferons, the interleukins, and TNF-α were traditionally considered to be unique to vertebrates. However, functional analogs of cytokines, such as IL-1, IL-2, IL-6, and TNF-α have been identified in different groups of invertebrates (e.g. annelids, tunicates, molluscs, echinoderms) (Ottaviani et al., 1996). These invertebrate-derived cytokine-like molecules have been shown to share many biological activities with vertebrate equivalents because of their conserved structure (Nappi and Ottaviani, 2000). In addition, a growing number of cytokines of shrimps have been identified in recent years. The TNF members have been identified in *Marsupenaeus japonicus*, *Litopenaeus vannamei*, and *Macrobrachium nipponense* (Mekata et al., 2010; Wang et al., 2012; Qin et al., 2019). The interleukin-1 receptor associated kinase-1 and interleukin-16-like gene were characterized in *Fenneropenaeus penicillatus* and *Litopenaeus vannamei* (Liang et al., 2017; Xu et al., 2017). The interferon regulatory factor was also identified in *Litopenaeus vannamei* (Li et al., 2015). The above studies might support indirectly that IL-1, IL-6, INF-γ, and TNF-α are also found in *M. rosenbergii*, and these cytokines could be good indicators to assess the effect of lipid sources on shrimp health as they are the main effectors of immune responses.

In the present study, contents of TNF-α, INF-γ, IL-1, and IL-6 significantly decreased in DSO compared with that in DFO, the same as IL-6 and TNF-α in DRO. It is postulated that proinflammatory cytokines production could be induced by oxidative stress which was caused by fish oil (Naik and Dixit, 2011), and the lipid peroxidation could be the main reason of the oxidative stress. Similar results were also found in common carp (*Cyprinus carpio*) (Chen X. M. et al., 2017), large yellow croaker (*Larmichthys crocea*) (Li et al., 2013), and rat (*Rattus norvegicus*) (Sanders et al., 2004). Additionally, considerable research supports that n-3 PUFA can induce lipid peroxidation (Pupe et al., 2002; Muralidhar et al., 2004). However, numerous studies have clearly described that n-3 LC-PUFA reduced the expression of inflammation-related genes in vertebrate macrophages, which caused a decreased proinflammatory cytokines production (Weldon et al., 2007; Mullen et al., 2010). However, little literature is available on shrimp, and the contradiction might be due to dietary levels of n-3 LC-PUFA. According to previous research, the anti-inflammatory effects induced by n-3 LC-PUFAs depend on the stimulation of macrophages, while macrophages can be induced by an appropriate type and dose of stimulants (Mullen et al., 2010; Forlenza et al., 2011). Therefore, the unconspicuous anti-inflammatory effects of n-3 LC-PUFAs in the present study could be due to the short of stimulating start-up process.

In the present study, prawns fed vegetable oils showed significantly lower iNOS and ASA activities, as well as NO contents than those fed fish oil. NO might behave as a potential antioxidant agent relying on its ability to eliminate oxygen free radicals. Previous *in vitro* studies support this concept in as much as NO is able to inhibit lipid peroxidation (Violi et al., 1999). Similar results were found in *Catla catla* (Sharma et al., 2017), mussel (Belavgeni and Dailianis, 2017) and *Litopenaeus vannamei* (Duan et al., 2017). Cytokines, especially TNF-α, are produced during liver injury, subsequently activate iNOS and induce NO release (Carnovale et al., 2000). In general, the activation of transcription factors NF-κB is an indispensable step for iNOS activity increasing, afterward inducing the release of NO (Pautz et al., 2010). In the present study, the expression levels of two major homologs of the components of NF-κB signal pathways, IMD-Relish and Toll-Doral, were both affected by the dietary lipid sources. The mRNA expression of the key gene in NF-κB signal pathway deceased significantly when fish oil was replaced by vegetable oils. It is speculated that both IMD-Relish and Toll-Dorsal signal pathway were activated by oxidative stress, which further caused lipid peroxidation in DFO. This conclusion supported by previous research that shows that oxidative stress is a major factor leading to the phosphorylation of IκB, that subsequently releases NF-κB, which than is translocated to the nucleus to regulate the expression of target genes (Bowie and O’Neill, 2000).

## Conclusion

**FIGURE 7:**
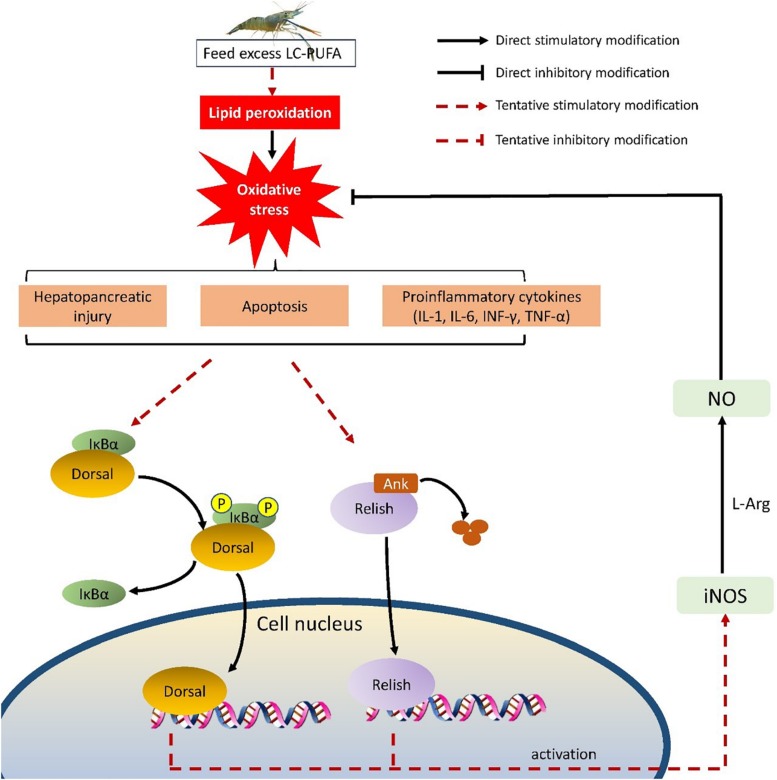
NF-κB/NO signaling mechanism in response to different lipid sources.

This present study investigated the growth performance and oxidative status of *M. rosenbergii* fed different lipid sources, and revealed the molecular regulatory mechanism in response to n-3 LC-PUFA. In the present study, vegetable oils replacing dietary fish oil benefited the growth performance, it could be attributed to the modified hepatopancreas oxidative status of prawns fed with vegetable oils, of which the fatty acid profile is different from fish oil. A putative mechanism to explain our results is shown in Figure 7. Excess n-3 PUFA causes lipid peroxidation, which exposes to oxidative stress. This leads to increased levels of hemolymph proinflammatory cytokines (TNF-α, INF-γ, IL-1, and IL-6), then hepatopancreatic cellular damage, and apoptosis occur. NF-κB signal pathway is activated to induce iNOS expression, which would induce NO production. NO would remit the oxidative stress. Thus, vegetable oils, such as soybean oil and RO might be the better lipid sources for *M. rosenbergii* than fish oil, which might be attributed to modified oxidative status induced by NF-κB-NO signal pathway.

## Data Availability Statement

The authors declared that the raw data underlying the conclusions made in the manuscript will be made available to other researchers by request.

## Ethics Statement

This study has been reviewed and approved by the Committee of the Animal Research Institute of Nanjing Agricultural University, China [permit number: SYXK (Su) 2011-0036]. The care and use of animals followed Animal Research Institute Committee guidelines of Nanjing Agricultural University, China.

## Author Contributions

CS contributed in the areas of experimental design, sampling, data analysis, and write-up. BL and QZ contributed to the experimental design and manuscript review. ZX and FS contributed to feeding and cultivating of experimental shrimps, sampling and statistics. HZ contributed to feed production, feeding of shrimps and sampling.

## Conflict of Interest

The authors declare that the research was conducted in the absence of any commercial or financial relationships that could be construed as a potential conflict of interest.

## Abbreviations

AA: arachidonic acid
ASA: anti-superoxide anion
DFO: group fed the diets included 6% fish oil
DRO: group fed the diets included 6% rapeseed oil
DSO: group fed the diets included 6% soybean oil
EFA: essential fatty acids
EPA: eicosapentaenoic acid
FCR: feed conversion ratio
FO: fish oil
IL-1: interleukin-1
IL-6: interleukin-6
IMD: immune deficiency
INF- γ: interferon- γ
iNOS: inducible-type NO-synthase
LA: linoleic acid
LC-PUFA: long-chain polyunsaturated fatty acids
LNA: linolenic acid
OA: oleic acid
RFI: relative feed intake
RO: rapeseed oil
SGR: specific growth rate
SO: soybean oil
TDT: terminal deoxynucleotidyl transferase
TNF- α: tumor necrosis factor- α
TUNEL: transferase-mediated dUTP-biotin nick end labeling
WGR: weight gain rate.
